# Make the Foundations Firm

**Published:** 1889-11

**Authors:** G. W. Decamp


ARTICLE VIII.

### MAKE THE FOUNDATIONS FIRM.

DR. G. W. DECAMP.

If a young man persevere, I ask not the measure of his brain. He has a sure passport to success. If the heavens should fall, they would find him on the way to mental excellence. Throw away the false idea that "circumstances make the man." Man is the builder of his own fortune. There are no beds of ease on which to carry drones to victory. Difficulties must come, as smoke flies upward; that is by a law of nature. They are blessings; they stimulate to greater action, and teach the mind to conquer. By them its powers are developed.

What makes the Indian swift on foot, but the fleetness of the deer? The eye of the hunter is keen, because of the shrewdness of the game. The weight of the hammer gives strength to the blacksmith's arm. Who are the deepest reasoners, but those who have dared to wrestle with great difficulties. What would the foal of the racer know of its power, if kept with the common herd, destined for the yoke: Difficulties arouse to action. Actions make the man.

That the mind may grow, it must be nourished. Man would not thrive if fed on apples. He needs a variety. The sciences are the food of the mind. Restrict it to any one, and it will become weak. The eye of the criminal confined to his cell is not as strong as that of the sailor, who looks from the mast head.

It is not only necessary for all who intend to practice the science of dentistry to be learned in what directly pertains to it, but they must be *educated.*

Some may object, by saying that it is not necessary. They will tell me our fathers were not learned; that some of the brightest stars of the present entered the profession illiterate; and, with a great degree of satisfaction, fold their hands for a *little more sleep.* Thus would they block the wheels of progress, and be banished to everlasting ignorance. Should we live in log huts because our fathers did? Should we refuse to improve our advantages because our fathers had them not? No! No! In the name of our God we cry, No! Deny me any pleasure, but deny me not the use of what I have and what I can get by my best endeavor. Talent is adverse to ignorance. Our advantages are greater than our fathers'; and tho some of them, by herculean efforts, stand among the clouds, many worship at the shrine of ignorance. Our fathers improved what they had; we should do likewise. If we do, we shall be *better* than *they.* We must be *wiser* or *more ignorant.*

The position and respect with which the title of D. D. S. is intended to command should urge a thorough education. If we respect ourselves we must aim at mental and moral excellence above our patients. Our land is covered with colleges, where the young may learn to think. There is no excuse for ignorance, when good books, the products of the richest minds and the labor of many years, are within the reach of all. If a thorough preparation is made, those who enter our profession may bless it, bless humanity, and be blest of God.

That we may be, *Resolved*, That we individually recognize it to be our duty to earnestly constrain all who intend to enter our profession to educate themselves, so they may be prepared to pursue a regular course of study in some dental college, in connection with private instruction. This is my hope.—*Dental Register.*

---

